# Outcome of Skeletal Reconstructive Surgery for Metastatic Bone Tumours in the Femur

**DOI:** 10.5704/MOJ.1703.013

**Published:** 2017-03

**Authors:** NH Mohamed-Haflah, Y Kassim, I Zuchri, W Zulmi

**Affiliations:** Department of Orthopaedics, Universiti Kebangsaan Malaysia, Cheras, Malaysia; * Department of Orthopaedics, Prince Court Medical Centre, Kuala Lumpur, Malaysia

**Keywords:** complications, functional outcome, quality of life, reconstructive surgery, skeletal metastasis

## Abstract

**Introduction:**

The role of surgery in skeletal metastasis is to reduce morbidity and improve the quality of life in terminally ill patients. We report our experience with patients who underwent skeletal reconstructive surgery for metastatic bone tumour of the femur.

**Materials and Methods:**

Twenty nine operations for skeletal metastasis of the femur performed in our centre between 2009 and 2015 were included in this study. We evaluated the choice of implant, complications, survival rate and functional outcome. Fourteen patients were still alive at the time of this report for assessment of functional outcome using Musculoskeletal Tumour Society (MSTS) form.

**Results:**

Plating osteosynthesis with augmented-bone cement was the most common surgical procedure (17 patients) performed followed by arthroplasty (10 patients) and intramedullary nailing (2 patients) There were a total of five complications which were implant failures (2 patients), surgical site infection (2 patients), and site infection mortality (1 patient). The median survival rate was eight months. For the functional outcome, the mean MSTS score was 66%.

**Conclusion:**

Patients with skeletal metastasis may have prolonged survival and should undergo skeletal reconstruction to reduce morbidity and improve quality of life. The surgical construct should be stable and outlast the patient to avoid further surgery.

## Introduction

The incidence of cancer is increasing worldwide^1^,^2^. In the USA there was an estimated 1.6 million new cancer cases in 2016^2^. This is attributed to advances not only in medical cancer treatment but also in techniques of screening and tumour detection, both of which have resulted in earlier diagnosis and treatment^3^^-^^9^. Consequently, the incidence of bone metastasis has also increased^4^^,^^9^. In the USA, the incidence of metastatic bone tumour in 2008 was approximately 300,000 adults^10^.

Skeletal metastases cause bone pain and pathologic fractures, both of which ultimately increase morbidity and reduce quality of life in patients with an already shortened life span^4^,^11^^-^^14^. Thus, the aim of treatment is to reduce pain and improve mobility with minimal complications. Nonoperative treatment in pathological fracture is inadequate due to poor healing rate compounded by the use of palliative radiotherapy ^14^^-^^16^. Previous studies have shown surgery improves pain, function and quality of life ^17^^-^^20^. Life expectancy for patients with bone metastasis ranges from a few weeks to many years. When contemplating surgery, several considerations must be taken into account. Firstly, recovery of the patient from surgery should be shorter than the expected survival duration^21^. Secondly, the construct used for fracture stabilization must be durable and have low mechanical failure rate to last the entire lifetime of the patient^4^,^22^.

The aim of our study was to evaluate the types of reconstruction used in our patients with metastatic bone tumour in the femur focusing on complications, functional outcome and survival rates.

## Materials and Methods

We identified 29 patients who underwent skeletal reconstruction for metastatic tumour of the femur from 1st January 2009 to 31st July 2015. Breast carcinoma was the most common primary tumour involving 15 patients (52%). This was followed by carcinoma of lung (4 patients), thyroid (2), colon (2), renal cell (2), prostate (2), cervical (1) and endometrium (1). The indications for surgery were the presence of pathological fracture or an impending fracture based on the Mirels scoring system.

The medical records of all the patients including all imaging investigation were reviewed to evaluate survival rate, choice of implant, surgical related complications and functional outcome. Failure of the implants or endoprosthesis reconstruction was defined as revision of any or all components of the implant, removal of the prosthesis or amputation of the limb.

## Results

Of the 29 patients, there were six males (21%) and 23 females (79%). The mean age of patients at the time of surgery was 61 years old. The most common site of involvement was the proximal femur (21 patients). Amongst these, 10 had lesions involving the neck of the femur and 11 had lesions in the subtrochanteric region. The other locations of metastasis were the diaphysis (6 patients) and one in the metaphyseal distal femur. One patient had multiple sites of involvement.

In the neck of femur group, arthroplasty was performed in all ten patients. The type of prosthesis used were bipolar hemiarthroplasty (5 patients), endoprosthesis (4 patients) and total hip arthroplasty (1 patient). Of the five patients who underwent bipolar hemiarthroplasty, three had extension of the disease to the intertrochanteric region involving the greater trochanter. Bipolar hemiarthroplasty was chosen instead of endoprosthesis due to cost issues. In these patients, cement was used to augment the neck. An opening was created in the subtrochanteric region for attachment of the abductor tendon using strong sutures. One patient with metastatic lesion in the neck of femur and acetabulum underwent total hip replacement. Four patients underwent tumour excision and reconstruction with endoprosthesis. We used polyprolene mesh wrapped around the endoprosthesis during soft tissue reconstruction.

**Fig. 1:**
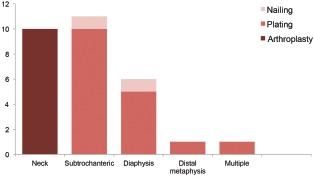
Methods of skeletal reconstruction according to sites of metastasis.

Osteosynthesis was performed in all 11 patients with metastatic disease in the subtrochanteric region, plating in 10 patients and intramedullary nailing in one patient. Intraoperatively, tumour curettage was performed to reduce the tumour load. We used two plates and augmentation with cement to ensure rigid fixation. For the patient who underwent intramedullary nailing, proximal femoral nail was inserted percutaneously without removing the tumour tissue. In the diaphysis group, double plating with cement augmentation was performed in five patients and intramedullary nailing in one patient. In the distal femur metaphysis group, all three patients underwent plating osteosynthesis. One patient had two sites of involvement: the subtrochanteric region and diaphysis. Double plating was performed proximally and distally. In total, for metastatic disease in the proximal femur, we performed 17 plating osteosynthesis, 10 arthroplasty and two intramedullary nailing (Fig. 1).

There were five patients who developed surgical complications two cases of implant failure, two cases of deep surgical site infection, and one intra-operative death. There were no instances of dislocation in any of our patients who had prosthetic replacement. One patient with breast carcinoma who underwent proximal femoral plating for subtrochanteric fracture femur sustained a periprosthetic fracture after a fall. We performed revision surgery using bipolar proximal femoral endoprosthesis. She later developed deep surgical site infection which was successfully treated with surgical debridement and intravenous antibiotics. The other implant failure occurred in a renal cell carcinoma patient who underwent proximal femoral plating for subtrochanteric fracture of the femur. She, however, refused any further surgical intervention. Intraoperative death occurred in a patient who underwent endoprosthesis of the proximal femur. Death was possibly due to a combination of heavy blood loss and cardiogenic effect of cement.

There were two cases of surgical site infection, both in patients with underlying breast carcinoma. One was in a patient who had plating of the midshaft femur. She later underwent hip disarticulation due to overwhelming infection. The other was a patient who sustained fracture neck of femur and underwent total hip replacement. She had multiple wound debridement and died one year later due to progression of the disease.

Data regarding postoperative radiotherapy was available in 28 patients. There were twenty four patients received radiotherapy. The patient who underwent total hip replacement did not receive radiotherapy due to unresolved deep surgical site infection. One patient died intraoperatively whilst another died before receiving radiotherapy. One patient refused any postoperative treatment.

**Fig. 2:**
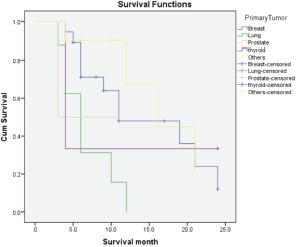
Kaplan-Meier survival rate by primary tumour.

Median survival time was eight months. Breast cancer patients had significantly longer survival than other primaries (Fig 2), with a mean survival rate of 14 months; the longest being 24 months. Patients with lung cancer had the shortest survival times (range 3-10 months). Amongst 14 patients who were still alive at the time of the study the mean MSTS score was 66%.

## Discussion

The goals of surgery in metastasis to long bones are to achieve local control and to stabilize the fracture or reconstruct the bony defect. Several methods of skeletal reconstruction have been employed for metastatic lesions of the femur, including endoprosthesis, intramedullary nailing and plating. The implant or prosthesis used for reconstruction must be durable with low mechanical failure rates to last the entire lifetime of patients with metastasis. In our series, the maximum length of survival was two years in a patient with breast cancer. It is essential that mechanical failures were avoided and complications minimized to prevent secondary revision surgery.

**Fig. 3:**
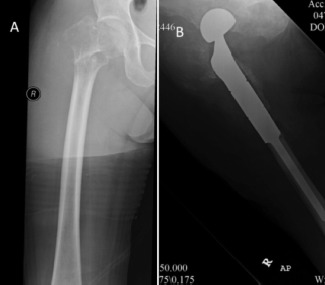
(A) Radiograph of a patient with metastatic disease of the proximal femur involving the intertrochanteric region. (B) Postoperative radiograph.

**Fig. 4:**
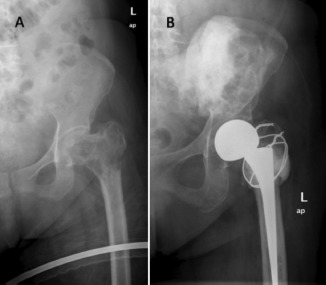
(A) A similar patient as in Figure 1. (B) Due to financial constraint reconstruction of the defect was performed with a bipolar hemiarthroplasty with cement augmentation and reattachment of the greater trochanter to the construct to preserve the abductor mechanism.

In the femoral head and neck region, local tumour resection with endoprosthetic reconstruction has been reported to provide good pain relief and functional outcome, thus, reducing complications associated with prolonged immobility ^5^,^23^-^28^. In addition, modular endoprosthesis used in primary neoplasia with a reported 10 years survivorship rate of 60-70%, should outlast patients with skeletal metastasis ^19^,^29^,^30^. When choosing the type of prosthesis, our protocol was to use endoprosthesis when the metastatic disease had extended distally into the inter-trochanteric region with involvement of the greater trochanter (Fig. 3). Furthermore, due to the design of endoprosthesis, wider tumour resection could be performed enhancing local tumour control. This would not be possible when reconstructing with bipolar hemiarthroplasty since to ensure joint stability the abductor mechanism including the greater trochanter must be preserved. However, some patients could not afford endoprosthesis and funding through local welfare system required a minimum of three months for approval. Thus, for these patients we opted for bipolar hemiarthroplasty with reconstruction of the neck region with cement and strong sutures for reattachment of the abductors (Fig. 4). There were no episodes of dislocation in our series.

**Fig. 5:**
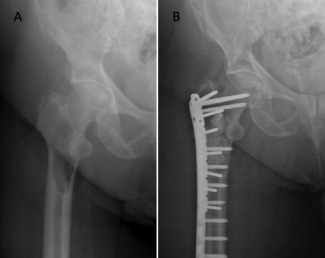
(A) Radiograph of a patient with metastatic disease of the proximal femur involving the subtrochanteric region. (B) Post-operative radiograph.

Controversy regarding the choice of implant for reconstruction arises in lesions or fractures in the metadiaphyseal region. In the proximal femur, Wedin *et al* in their series found that the failure rate in fractures treated with osteosynthesis was 16% compared to 9% in those treated with endoprosthesis^24^. Harvey *et al* reported a mechanical failure rate of 11% and 0% in the osteosynthesis and endoprosthesis group, respectively^31^. Mechanical failures were either due to non- union or progression of disease and lead to secondary surgery which was more complex with a higher risk of complications. However, Ramakrishnan *et al* found no incidence of mechanical failure in their series of pathological subtrochanteric fractures treated with proximal femoral nail^32^. In our series, all 11 patients who presented with metastatic lesion in the subtrochanteric region underwent osteosynthesis. Of these 11 patients, plating was performed in all but one case. Although we agree with previous authors regarding the superiority of endoprosthesis over osteosynthesis in the proximal region, plates remain our choice of implant due to cost factor as mentioned earlier. We advocate double plating with cement augmentation for a rigid fixation to allow immediate weight-bearing (Fig. 5).

In the diaphyseal region, intramedullary nailing provides effective resistance against angulation, torque and distraction forces^33^. It offers stable fixation of the whole bone and can be inserted percutaneously reducing complications associated with extensive tissue dissection. However, complications such as embolisation has been reported with reaming or long stemmed prosthesis^34^-^36^. Similar to the subtrochanteric region, plating was our choice of skeletal reconstruction in the diaphyseal region. We placed great importance in reducing tumour burden to prevent tumour progression, necessitating extensive exposure and incurring potential heavy bleeding. Failure to remove the tumour during percutaneous nailing and potential seeding of tumour cells in the canal during reaming may cause progression of disease leading to failure of implant. Wedin *et al* in their series found a higher rate of failure in patients who underwent curettage only (19%) compared to those who had local excision of tumour and cementation (11%)^24^. In our series, we performed tumour curettage only and not local excision. Our preference for plating once the tumour had been removed is because the use of intramedullary nail would require another entry point and also potential seeding of tumour cells during reaming. Furthermore, with the advent of locking plates, the locking mechanism of the screws can prevent screw pullout and subsequent mechanical failure^37^. Our complication rates were comparable to other previous studies (Table I). Another advantage of intramedullary nailing is the ability to address the whole length of the femur, thus, potentially avoiding the occurrence of periprosthetic fracture from residual disease. In our series, there were no incidence of periprosthetic fractures in those who underwent plating in the diaphyseal region. The two patients who sustained periprosthetic fractures had lesions in the subtrochanteric region and were not suitable for nailing.

**Table I:** Complications reported by other centres

	Site	No	Infection	Implant Failure	Dislocation
Wedin^24^, 2005	Femur	145	3%	10%	13%
Ahlmann^23^, 2006	Lower Limb	211	5.2%	10%	1.4%
Nillson27, 2007	Femur	245	1%	2%	5%
Seo^38^, 2010	Lower Limb	13		7%	
Harvey^31^, 2012	Femur	159	10%	Nil	10%
			2%	13%	
Sorenson^5^, 2013	Extremities	140	2%	<1%	8%
UKM series		42	5%	10%	Nil

Aside from location of the lesion, prognostic factor must also be taken into account when deciding on the appropriate construct. Postoperative length of survival is an important factor in the occurrence of mechanical failure^39^. In patients with good prognosis and longer expected survival time, local resection of tumour is advocated^24^,^25^,^27^. Wide local resection of solitary metastatic lesion in renal carcinoma has been reported to give good outcome with low recurrence rate^40^. However, surgical intervention is not necessarily excluded in a patient with a predicted short lifespan since one can never know with absolute certainty the exact survival time of any terminal patient. Thus, in our centre, we advocate surgical intervention in all patients who are fit to undergo surgery.

Irrespective of the type of skeletal reconstruction employed the main objective in the treatment of patients presenting with skeletal metastasis is to improve quality of life. Clohisy *et al* stated the difficulty in performing researches to investigate the impact of surgery in skeletal metastasis on quality of life^41^. Most studies have a heterogeneous group of patients with different treatment, medical and surgical, for their primary tumour. Similarly, our study had the same limitations. In our series the MSTS score was 66% which is deemed satisfactory. It was essential that our construct was stable to allow immediate weight bearing thereby reducing immobility related complications and improving quality of life. Harvey et *al* in their series of proximal femur metastasis reported an MSTS score of 80% in patients with intramedullary devices and 70% in patients with endoprosthesis^31^. In the distal femur, Ahlmann et *al* reported a score of 74% in their series of patients who underwent endoprosthetic reconstruction^23^.

In conclusion, patients with skeletal metastasis to the femur survive, on average, up to one year and benefited from skeletal reconstruction. Treatment should be aimed at improving their quality of life by reducing pain and improving mobility and function. Post-operative complications such as implant failure and infection should be minimised to avoid secondary surgery which would increase morbidity in such patients. Arthroplasty and osteosynthesis with cement augmentation produced good functional outcome with minimal risk of failure.

## Conflicts of Interest

The authors declare no conflicts of interest related to the subject matter or materials discussed in this article.
